# The Role of Approximate Number System in Different Mathematics Skills Across Grades

**DOI:** 10.3389/fpsyg.2018.01733

**Published:** 2018-09-18

**Authors:** Dan Cai, Linni Zhang, Yan Li, Wei Wei, George K. Georgiou

**Affiliations:** ^1^College of Education, Shanghai Normal University, Shanghai, China; ^2^Department of Educational Psychology, University of Alberta, Edmonton, AB, Canada

**Keywords:** approximate number system, non-symbolic estimation, symbolic estimation, mathematics skills, Chinese

## Abstract

Although approximate number system (ANS) has been found to predict mathematics ability, it remains unclear if both aspects of ANS (symbolic and non-symbolic estimation) contribute equally well to mathematics performance and if their contribution varies as a function of the mathematics outcome and grade level. Thus, in this study, we examined the effects of both aspects of ANS on different mathematics skills across three grade levels. Three hundred eleven children (100 children from kindergarten, 107 children from Grade 2, and 104 children from Grade 4) from two kindergartens and three elementary schools in Shanghai, China, were assessed on measures of ANS (dot estimation and number line estimation), general cognitive ability (nonverbal intelligence, inhibition, and working memory), and mathematics abilities (numerical operations and mathematical problem solving in all grades, early mathematical skills in kindergarten, and calculation fluency in Grades 2 and 4). Results of hierarchical regression analyses showed that, in kindergarten, non-symbolic estimation predicted all mathematics skills even after controlling for age, gender, and general cognitive ability. In Grades 2 and 4, symbolic estimation accounted for unique variance in mathematical problem solving, but not in calculation fluency. Symbolic estimation also predicted numerical operations in Grade 4. Taken together, these findings suggest that in the early phases of mathematics development different aspects of ANS contribute to different mathematics skills.

## Introduction

The approximate number system (ANS) is a mental system responsible for representing and processing numerical magnitude information (De Smedt et al., 2013; Libertus, 2015). It has been argued that ANS helps children form imprecise numerical estimations that are later on activated and used in magnitude comparisons (Siegler and Lortie-Forgues, 2014) and in mathematics learning (see Clements and Sarama, 2007; Feigenson et al., 2013; Libertus, 2015; Mussolin et al., 2016, for reviews). However, far less is known about the conditions under which the two most known ANS aspects (symbolic and non-symbolic estimation) predict mathematics skills. Therefore, this study aimed to examine how the two ANS aspects (symbolic and non-symbolic estimation) contribute to different mathematics skills (early mathematics skills, numerical operations, mathematical problem solving, and calculation fluency) in different grade levels (kindergarten, Grade 2, and Grade 4).

Approximate number system consists of two aspects: non-symbolic estimation and symbolic estimation. Non-symbolic estimation refers to the processing of quantities and numerosities without using numerals (Smets et al., 2015). It emerges as early as at the age of 6 months, when infants discriminate between large ratios of two arrays (e.g., 6:12; Libertus and Brannon, 2010), and continues to develop until adulthood, when individuals use this knowledge to discriminate between smaller ratios (e.g., 0.9:1; Price et al., 2012). In turn, symbolic estimation refers to mapping the numerals on a quantitative dimension, such as approximating the number of dots in a picture and the location of a number on a number line (Booth and Siegler, 2006). It is hypothesized that the numerals are mentally represented along a mental number line (Siegler and Lortie-Forgues, 2014) and the representations of numerals become more accurate from a logarithmic manner to a linear manner as children get older (Siegler and Booth, 2004; Friso-van den Bos et al., 2015). Meta-analyses have reported significant correlations between the two ANS aspects and mathematics (Chen and Li, 2014; Fazio et al., 2014; Schneider et al., 2018a). For example, Chen and Li (2014) estimated the average correlation between non-symbolic estimation and mathematics to be 0.24, and Schneider et al. (2018a) reported an average correlation between symbolic estimation and mathematics of 0.44.

The meta-analyses, however, have also detected great heterogeneity among the correlations. A possible explanation for this heterogeneity may be that the two ANS aspects exert a different effect on mathematics skills in different grades. To delineate this, a study should examine the role of both ANS aspects in mathematics across different grade levels (what we did in our study). Besides, it is also possible that the effects of grade level interact with the type of mathematics skill assessed in different studies. Mathematics skills include a wide range of skills such as early mathematics skills (e.g., counting and number knowledge), numerical operations (i.e., the ability to use algorithms to solve written arithmetic), calculation fluency (the ability to retrieve arithmetic facts from memory quickly), and mathematical problem solving (the ability to apply mathematical concepts and arithmetic to solve contextual problems). Some researchers (Libertus et al., 2013; Wang et al., 2016) have argued that non-symbolic estimation may help children learn number-related knowledge such as number concepts, number intrarelationships, and thus be more important for early mathematics abilities. In later years, symbolic estimation may help children understand symbolic arithmetic and facilitate recall of answers to arithmetic problems (Siegler and Braithwaite, 2017), and thus be more important in mathematics in later grades. Recently, Tosto et al. (2017) also argued that once arithmetic skills become automatized, neither non-symbolic nor symbolic estimation should play an important role. This should particularly affect calculation fluency since children (particularly Chinese)^1^ become efficient in executing simple calculations as early as in Grade 1 (e.g., Deng et al., 2015; Cui et al., 2017).

Only a few studies have also contrasted the effects of both symbolic and non-symbolic estimation in the same study (e.g., Sasanguie et al., 2012, 2013; Jordan et al., 2013; Lyons et al., 2014; Cirino et al., 2016; Tosto et al., 2017). Most of these studies have shown that number line estimation uniquely explains mathematics skills after controlling for non-symbolic estimation (e.g., Sasanguie et al., 2012; Jordan et al., 2013; Lyons et al., 2014; Cirino et al., 2016; Tosto et al., 2017), but none of these studies have examined how the two ANS skills explain early mathematics skills. Although non-symbolic estimation appears to be less important in learning mathematics in school years, as reviewed before, it may uniquely explain mathematics skills in early years.

Interestingly, most of the previous studies examining the role of ANS in mathematics did not control for the effects of key cognitive predictors of mathematics such as nonverbal intelligence or executive functioning. Executive functioning, the cognitive skills engaged in goal-directed activities, includes inhibition and working memory (e.g., Miyake et al., 2000; Lehto et al., 2003), both of which are significant correlates of mathematics skills (e.g., Swanson, 2006; Bull et al., 2008; Lan et al., 2011; Cragg et al., 2017; see Bull and Lee, 2014, for a review). Executive functioning may also contribute to non-symbolic and symbolic estimation (e.g., Xenidou-Dervou et al., 2013; Wong et al., 2016; Peng et al., 2017; Zhu et al., 2017; Purpura and Simms, 2018). Inhibition may be required in suppressing non-numerical stimulus features and focus attention on the magnitude (Starr et al., 2017), and working memory may be needed in holding symbolic or non-symbolic information in rapid comparison of two arrays of objects (Xenidou-Dervou et al., 2013) and in holding the bounds or referent points and their corresponding values in number line tasks (Schneider et al., 2018b). Therefore, the association between ANS acuity and mathematics may be accounted for by executive functioning. Price and Wilkey (2017), for example, found that inhibition and working memory partly mediated the relationship between ANS acuity (both non-symbolic and symbolic estimation) and mathematics skills.

Notice also that most previous studies on ANS were conducted in Western countries and far less is known about the role of ANS acuity in learning mathematics in East Asian countries (e.g., China). The place-value system in Chinese is relatively transparent (e.g., “
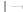
(ten-one)” for eleven), which may facilitate Chinese children learning symbolic numbers (Miller et al., 2005). The easier mastery of symbolic numbers in Chinese may result in non-symbolic estimation being less important in learning mathematics. There are reasons to believe that non-symbolic and symbolic estimation may play a different role in China than in Western countries. To date, only a handful of studies have examined the effects of symbolic or non-symbolic estimation on mathematics in Chinese children (see Lonnemann et al., 2011; He et al., 2016; Wang et al., 2016; Wong et al., 2016; Zhang et al., 2016; Cui et al., 2017; Peng et al., 2017; Zhu et al., 2017), and none of these studies have examined how symbolic and non-symbolic estimation predict different mathematics skills in both early and later elementary school years.

Therefore, the present study aimed to examine the effects of both ANS aspects (symbolic and non-symbolic estimation) on different mathematics skills (early mathematics skills, numerical operations, mathematical problem solving, and calculation fluency) in different grade levels in China. Based on the findings of previous studies (Jordan et al., 2013; Lyons et al., 2014; Wong et al., 2016; Tosto et al., 2017; Zhu et al., 2017), we hypothesized that:

1) The effects of symbolic and non-symbolic estimation will vary as a function of grade level. Non-symbolic estimation will uniquely predict mathematics skills only in kindergarten, while symbolic estimation will uniquely predict mathematics skills at all grade levels.
2) Non-symbolic and symbolic estimation will predict different mathematics skills. Non-symbolic estimation will predict early mathematics skills, that is counting, symbolic number knowledge and arithmetic, and symbolic estimation will predict all mathematics skills except from calculation fluency.

## Materials and Methods

### Participants

The participants were 100 children from kindergarten (53 girls and 47 boys; mean age = 66.53 months, *SD* = 3.31), 107 children from Grade 2 (60 girls and 47 boys; mean age = 92.16 months, *SD* = 3.96), and 104 children from Grade 4 (59 girls and 44 boys; mean age = 115.75 months, *SD* = 3.62). The children were recruited on a voluntary basis from two kindergartens and three elementary schools in Shanghai, China. The schools that participated in our study serve primarily middle-class families and the demographics are representative of the general population in Shanghai (The National Bureau of Statistics in Shanghai, 2017). All children were native Mandarin speakers and none was diagnosed with any intellectual, sensory, or behavioral disorders. Parental consent and ethics approval from the Shanghai Normal University were obtained prior to testing.

### Materials

#### General Cognitive Abilities

##### Nonverbal intelligence

Nonverbal Matrices from Cognitive Assessment System-Version 2 (CAS-2; Naglieri et al., 2014), was used to assess nonverbal intelligence. Children were presented with a variety of geometric designs that were missing one part and were asked to select the missing part among six options. The task was discontinued after four consecutive errors. The score was the total number correct (max = 44). Criterion validity has been reported to range from 0.57 to 0.65 (Naglieri et al., 2014). The Cronbach’s alpha reliability coefficient in the current study was 0.85 in Kindergarten, 0.91 in Grade 2, and 0.90 in Grade 4.

##### Executive functioning

###### Inhibition

Expressive Attention, adopted from CAS-2 (Naglieri et al., 2014) was used to assess children’s inhibition. Two versions (5–7 years and 8–18 years) were used to avoid ceiling/floor effects. The version used for children in Grades 2 and 4 is similar to the color-word Stroop test (Stroop, 1935) and includes three pages. In the first page, children were asked to say aloud the names of color squares (e.g., blue, yellow, red, and green) and, in the second, children were asked to name the color characters (e.g., “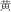,” yellow). In the third page, children were presented with 40 color characters each printed in a color different from the color character [e.g., “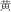 (yellow)” printed in blue ink]. They were asked to read aloud the color of the ink in which the characters were printed as quickly as possible. An 8-item practice trial was used to make sure children understood the instructions prior to testing. A ratio score was calculated by dividing the number of correct responses by the time to finish naming all 40 items. Criterion validity has been reported to range from 0.69 to 0.73 (Naglieri et al., 2014). The Cronbach’s alpha reliability coefficient in the current study was 0.86 in both Grades 2 and 4.

The version for 5–7 year old students was used in kindergarten and it also included three pages. In each page, children were shown animal drawings that included small animals (butterfly, mouse, bird, and frog) and big animals (elephant, whale, horse, and bear), and were asked to say aloud whether each animal was small or big as fast as they could. In the first page, animal drawings were printed in a uniform size, and in the second page, big animals were printed in big size and small animals in a small size. In the third page, big animals were printed in a small size and small animals in a big size, and children were asked to name the animal drawing based on their actual size and not based on the size they were printed. The score was the number of correct responses in the third page divided by the time to finish naming the items. Criterion validity has been reported to range from 0.51 to 0.67 (Naglieri et al., 2014). The Cronbach’s alpha reliability coefficient in the current study was 0.81.

###### Working memory

Digit Span Forward from CAS-2 (Naglieri et al., 2014) was used to assess children’s working memory. The test consists of 2–9 span with four trials in each span. The numbers were orally presented at the speed of one number per second and then children were asked to repeat these numbers in the same order. The test was discontinued when three errors were made in each span. The score was the final span that the children had reached. Criterion validity has been reported to range from 0.40 to 0.64 (Naglieri et al., 2014). The Cronbach’s alpha reliability coefficient in the current study was 0.88, 0.89, and 0.88 in Kindergarten, Grade 2, and Grade 4, respectively.

#### Mathematics Skills

##### Early mathematics skills

Test of early mathematics ability (TEMA-3; Ginsburg and Baroody, 2003) was used to measure kindergarteners’ early mathematics skills. TEMA-3 includes 72 items on counting, symbolic number knowledge, and arithmetic. The test was discontinued after four consecutive errors and the children’s score was the total number correct. TEMA-3 has been found to correlate significantly with other math tests such as Mathematics subtest of the Young Children’s Achievement Test and Key Math Revised (*r*’s range from 0.54 to 0.91; Ginsburg and Baroody, 2003). The Cronbach’s alpha reliability coefficient in the current study was 0.88.

##### Numerical operations

Numerical operations, adopted from WIAT-III (Wechsler Individual Achievement Test-Third Edition; Wechsler, 2009), was used to assess children’s numerical operations skills under untimed conditions. The items were arranged in increasing difficulty and children were asked to solve these items one by one. The test was discontinued after four consecutive errors and a participant’s score was the total number correct. Numerical operations has been found to correlate significantly with other math measures such as numerical operations in WIAT-II and Math Reasoning (*r*’s range from 0.71 to 0.81; Wechsler, 2009). The Cronbach’s alpha reliability coefficient in the current study was 0.80 and 0.89 in Grades 2 and 4, respectively.

##### Mathematical problem solving

Math problem solving, adopted from WIAT-III (Wechsler Individual Achievement Test-Third Edition; Wechsler, 2009), was used to assess mathematical problem solving. The items in the task were arranged in terms of increasing difficulty (max = 72). Children were asked to solve these items one by one, under untimed conditions. The test was discontinued after four consecutive errors and a participant’s score was the total number correct. Math problem solving has been found to correlate significantly with other math measures such as numerical operations and math reasoning (*r*’s range from 0.75 to 0.84; Wechsler, 2009). The Cronbach’s alpha reliability coefficient in the current study was 0.88, 0.90, and 0.90 in Kindergarten, Grade 2, and Grade 4, respectively.

##### Calculation fluency

Math fluency from WIAT-III (Wechsler Individual Achievement Test-Third Edition; Wechsler, 2009) was used to assess children’s calculation fluency. This task includes three subtests: addition fluency (e.g., 5 + 1 = 6), subtraction fluency (e.g., 4 - 2 = 2), and multiplication fluency (e.g., 2 × 3 = 6). Children were asked to write down the answers to 48 items in each subtest as soon as they could in 1-min time limit. A participant’s score was the sum of three subtests’ scores. Math fluency has been found to correlate significantly with other math measures such as numerical operations and math reasoning (*r*’s range from 0.55 to 0.64; Wechsler, 2009). Zhu et al. (2017) reported internal consistency reliability for math fluency to be 0.88 and 0.93 for Grades 2 and 4, respectively.

#### Approximate Number System

##### Non-symbolic estimation

Dot estimation, adapted from Halberda and Feigenson (2008), was used to assess non-symbolic estimation task on a computer. At the time of testing, two pictures would appear on the screen. There were some random points (10–30 points) on each picture. The number of points on the two pictures was different. In Grades 2 and 4, children were asked to judge which picture had more points within a 2 s time limit. In kindergarten, children were given 3 s to make a decision^2^. The task included 6 practice items and 24 test items. A participant’s score was the percentage of accurate responses across the 24 items. The task has been used in several previous studies in Chinese showing good psychometric properties (e.g., Cui et al., 2017; Zhu et al., 2017; Cheng et al., 2018). The Cronbach’s alpha reliability coefficient in the current study was 0.69, 0.77, and 0.72, for Kindergarten, Grade 2, and Grade 4, respectively.

##### Symbolic estimation

Number line estimation was adopted from Opfer and Siegler (2007) and was used to measure children’s symbolic estimation. The version for Grade 2 and Grade 4 was carried out on an 8-inch tablet. There was a line displayed on the pad 0 was marked on the left of the line, and 100 was marked on the right. At the time of testing, a number would appear on the screen, and children were asked to estimate which position this number was in 0–100 and mark the position on the line. The items included 26 numbers: 3, 4, 6, 8, 12, 17, 20, 21, 23, 25, 29, 33, 39, 43, 48, 50, 52, 57, 61, 64, 72, 79, 81, 84, 90, and 96. The items were presented in random order. In kindergarten, the number line task was given as a paper and pencil task. The actual length of the line was 24 cm and it was used to represent the distance from 0 to 10. The items included nine numbers: 1, 2, 3, 4, 5, 6, 7, 8, and 9. The formula to calculate the final score was: $\frac{\text{|Estimation}-\text{Estimation Quantity|}}{\text{Scale of Estimation}}$. The task has been used in previous studies in Chinese showing good psychometric properties (e.g., Siegler and Mu, 2008; Laski and Yu, 2014; Zhu et al., 2017). The Cronbach’s alpha reliability coefficient in our sample was 0.72, 0.80, and 0.69, for Kindergarten, Grade 2, and Grade 4, respectively.

### Procedures

Children were individually tested by trained graduate students in a quiet room in their school. The testing was completed in two sessions of 30–40 min each. Session A included the mathematics tests [math problem solving, numerical operations, math fluency (only in Grades 2 and 4), TEMA-3 (only in Kindergarten)]. Session B included the cognitive tests (nonverbal matrices, expressive attention, and digit span forward) and the ANS tasks (dot estimation and number line estimation). Half of the children in each grade level did first Session A and then Session B. The other half did the sessions in the reverse order.

## Results

### Preliminary Data Analyses

**Table 1** shows the descriptive statistics (mean, standard deviation, range, and kurtosis and skewness) for all the measures in our study. The distributions of numerical operations and dot estimation were positively skewed and thus log transformation was applied. After the log transformation, their distributions were normalized and the transformed scores were used in further analyses.

**Table 1:** Descriptive Statistics for all Measures Used in our Study.

	Kindergarten				Grade 2				Grade 4			
	M (*SD*)	Range	Skew.	Kurt.	M (*SD*)	Range	Skew.	Kurt.	M (*SD*)	Range	Skew.	Kurt.
NO	14.66 (4.98)	0–26	-0.78	0.96	20.83 (2.84)	16–30	1.14	1.18	33.41 (3.21)	24–52	2.11	11.48
NO (lg)	1.14 (0.17)	0.67–1.41	-1.46	1.60	1.32 (0.06)	1.2–1.5	0.78	0.55	1.52 (0.03)	1.45–1.63	0.92	1.79
MPS	30.52 (5.65)	13–41	-0.53	0.19	42.35 (4.67)	31–58	0.08	0.71	50.61 (3.52)	42–65	1.16	3.55
TEMA	42.79 (13.02)	16–69	-0.03	-0.69	∖	∖	∖	∖	∖	∖	∖	∖
MF	∖	∖	∖	∖	67.21 (14.07)	39–98	0.15	-0.76	106.79 (16.96)	71–136	-0.01	-0.78
DE	0.78 (0.13)	0.38–0.96	-1.00	0.69	0.73 (0.10)	0.52–0.96	0.10	-0.23	0.77 (0.11)	0.38–0.96	-1.23	2.58
NLE	0.19 (0.09)	0.04–0.59	0.96	3.15	0.07 (0.03)	0.02–0.21	1.58	3.70	0.06 (0.03)	0.02–0.26	3.60	19.49
NLE(lg)	-0.77 (0.23)	-1.44–(-0.23)	-0.80	0.51	-1.20 (0.18)	-1.69–(-0.69)	0.20	0.19	-0.26 (0.17)	-1.75–(-0.59)	0.62	2.99
Intelligence	18.44 (6.15)	1–32	-0.66	0.76	25.12 (5.00)	14–37	0.19	-0.45	26.91 (5.89)	16–39	0.36	-0.73
Inhibition	0.64 (0.17)	0.03–1.09	-0.16	1.72	1.25 (0.47)	0.37–2.50	0.02	-0.49	0.74 (0.19)	0.08–1.31	-0.10	1.46
WM	6.75 (1.46)	4–9	-0.05	-1.07	8.00 (1.03)	5–9	-0.81	-0.23	8.23 (0.90)	6–9	-0.97	0.10

*NO, numerical operations; MPS, math problem solving; TEMA, test of early mathematics ability; MF, math fluency; DE, dot estimation; NLE, number line estimation; Intelligence, nonverbal intelligence; WM, working memory.*

### Correlations Between the Measures

The correlation coefficients among all variables in kindergarten, Grade 2, and Grade 4 are presented in **Tables 2**, **3**. In kindergarten, both the number line estimation and dot estimation correlated significantly with all mathematics skills (*r*’s ranged from -0.43 to -0.55). In Grade 2, number line estimation correlated significantly with math problem solving (*r* = -0.52) and math fluency (*r* = -0.21). In Grade 4, number line estimation correlated significantly with math problem solving (*r* = -0.28) and numerical operations (*r* = -0.27). Dot estimation did not correlate significantly with any math task in Grades 2 and 4.

**Table 2:** Correlations between the variables in kindergarten.

	1.	2.	3.	4.	5.	6.	7.	8.	9.	10.
1. Math problem solving										
2. Numerical operation	0.73^∗∗^									
3. TEMA	0.84^∗∗^	0.68^∗∗^								
4. Number Line Estimation	-0.37^∗∗^	-0.28^∗∗^	-0.55^∗∗^							
5. Dot Estimation	0.40^∗∗^	0.37^∗∗^	0.43^∗∗^	-0.13						
6. Nonverbal Intelligence	0.68^∗∗^	0.63^∗∗^	0.62^∗∗^	-0.33^∗∗^	0.28^∗∗^					
7. Inhibition	0.33^∗∗^	0.37^∗∗^	0.34^∗∗^	-0.07	0.25^∗^	0.25^∗^				
8. Working Memory	0.37^∗∗^	0.22^∗^	0.36^∗∗^	-0.19	0.12	0.23^∗^	0.21^∗^			
9. Age	0.21^∗^	0.15	0.13	-0.15	0.13	0.15	0.21^∗^	0.08		
10. Gender	0.12	0.16	0.16	-0.18	-0.09	0.07	0.05	0.07	0.16	

*^∗^p < 0.05, ^∗∗^p < 0.01.*

**Table 3:** Correlations between the variables in Grade 2 (below the diagonal) and Grade 4 (above the diagonal).

	1.	2.	3.	4.	5.	6.	7.	8.	9.	10.
1. Math problem solving		0.56^**^	0.28^**^	-0.28^**^	-0.09	0.38^**^	0.21^*^	0.09	0.06	0.23^**^
2. Numerical operation	0.19^*^		0.22^*^	-0.27^**^	0.01	0.26^**^	0.27^**^	0.15	0.10	0.15
3. Math fluency	0.23^*^	-0.07		-0.09	-0.00	-0.05	0.24^*^	0.09	0.06	0.15
4. Number line estimation	-0.52^**^	-0.14	-0.21^*^		0.04	-0.21^*^	-0.08	-0.04	-0.08	-0.09
5. Dot estimation	0.15	0.10	0.05	-0.28^**^		-0.01	0.16	-0.02	0.16	0.04
6. Nonverbal intelligence	0.26^**^	0.10	0.01	-0.32^**^	0.04		0.14	-0.00	-0.02	0.18
7. Inhibition	0.27^**^	0.12	0.12	-0.26^**^	0.08	-0.05		-0.07	0.10	0.02
8. Working memory	0.29^**^	-0.08	0.21^*^	-0.25^*^	0.21^*^	0.28^**^	0.00		0.04	0.02
9. Age	0.16	0.12	0.12	-0.13	-0.04	0.18	0.25^**^	-0.03		0.00
10. Gender	0.07	0.16	0.03	-0.13	-0.12	0.07	-0.01	-0.08	0.18	

*^∗^p < 0.05, ^∗∗^p < 0.01.*

### Results of Regression Analyses

Hierarchical regression analyses were subsequently conducted within each grade level to examine the unique contribution of the two ANS aspects to mathematics outcomes [math problem solving, numerical operations, math fluency (assessed only in Grades 2 and 4), and TEMA (assessed only in kindergarten)]. In each model, age and gender were entered in the regression equation at step 1 as control variables. The general cognitive abilities (nonverbal intelligence, inhibition, and working memory) were entered in the regression equation at step 2, and number line estimation and dot estimation were entered at step 3 of the regression equation as a block.

**Tables 4**–**6** show the standardized beta coefficients, *R*^2^ changes, and significance levels of the regression models in each grade level. In kindergarten, the two ANS aspects accounted for unique variance in math problem solving [5%, but only dot estimation had a significant effect (β = -0.190, *p* < 0.01)], numerical operations [4%, but only dot estimation had a significant effect (β = -0.192, *p* < 0.05)], and TEMA-3 [17%, both number line estimation (β = -0.358, *p* < 0.001) and dot estimation (β = -0.246, *p* < 0.01) had a significant effect], after controlling for age, gender, nonverbal intelligence, inhibition, and working memory. In Grade 2, ANS accounted for unique variance in math problem solving [14%, but only the effects of number line estimation were significant (β = -0.444, *p* < 0.001)], but not in numerical operation and math fluency. In Grade 4, ANS accounted for unique variance in math problem solving [5%, but only the effects of number line estimation were significant (β = -0.184, *p* < 0.05)], but not in math fluency. The predictive effect of number line estimation on numerical operations was also significant (β = -0.203, *p* < 0.05).

**Table 4:** Results of hierarchical regression analyses predicting math problem solving in kindergarten, Grade 2, and Grade 4.

		Kindergarten		Grade 2		Grade 4	
Step		β	Δ*R*^2^	β	Δ*R*^2^	β	Δ*R*^2^
1.	Age	0.200^∗^	0.05	0.154	0.03	0.060	0.07^∗^
	Gender	-0.088		-0.030		-0.248^∗^	
2.	Nonverbal intelligence	0.592^∗∗∗^	0.49^∗∗∗^	0.187	0.17^∗∗∗^	0.340^∗∗∗^	0.16^∗∗^
	Inhibition	0.116		0.269^∗∗^		0.154	
	Working memory	0.205^∗∗^		0.235^∗^		0.099	
3.	Number line estimation	-0.121	0.05^∗∗^	-0.444^∗∗∗^	0.14^∗∗∗^	-0.184^∗^	0.05^∗^
	Dot estimation	0.190^∗∗^		-0.052		-0.131	
Total *R*^2^			0.59^∗∗∗^		0.34^∗∗∗^		0.28^∗∗∗^

*^∗^p < 0.05, ^∗∗^p < 0.001, ^∗∗∗^p < 0.001.*

**Table 5:** Results of hierarchical regression analyses predicting numerical operations in kindergarten, Grade 2, and Grade 4.

		Kindergarten		Grade 2		Grade 4	
Step		β	Δ*R*^2^	β	Δ*R*^2^	β	Δ*R*^2^
1.	Age	0.123	0.04	0.096	0.04	0.127	0.04
	Gender	-0.140		-0.143		-0.148	
2.	Nonverbal intelligence	0.555^∗∗∗^	0.41^∗∗∗^	0.127	0.03	0.231^∗^	0.14^∗∗^
	inhibition	0.212^∗^		0.124		0.240^∗^	
	Working memory	0.045		-0.098		0.157	
3.	Number line estimation	-0.054	0.04^∗^	-0.065	0.01	-0.203^∗^	0.04
	Dot estimation	0.192^∗^		0.093		-0.055	
Total *R*^2^			0.49^∗∗∗^		0.08		0.22^∗∗^

*^∗^p < 0.05, ^∗∗^p < 0.01, ^∗∗∗^p < 0.001.*

**Table 6:** Results of hierarchical regression analyses predicting TEMA and math fluency (MF) in kindergarten, Grade 2, and Grade 4.

		Kindergarten (TEMA-3)		Grade 2 (MF)		Grade 4 (MF)	
Step		β	Δ*R*^2^	β	Δ*R*^2^	β	Δ*R*^2^
1.	Age	0.103	0.04	0.121	0.02	0.021	0.02
	Gender	-0.148		0.004		-0.133	
2.	Nonverbal intelligence	0.525^∗∗∗^	0.43^∗∗∗^	-0.050	0.06	-0.151	0.09^∗^
	inhibition	0.170^∗^		0.098		0.271^∗∗^	
	Working memory	0.194^∗^		0.241^∗^		0.104	
3.	Number line estimation	-0.358^∗∗∗^	0.17^∗∗∗^	-0.148	0.02	-0.067	0.01
	Dot estimation	0.246^∗∗^		-0.002		-0.033	
Total *R*^2^			0.64^∗∗∗^		0.10		0.12^∗^

*^∗^p < 0.05, ^∗∗^p < 0.01, ^∗∗∗^p < 0.001.*

## Discussion

The aim of this study was to examine how two ANS aspects (symbolin) predict different mathematics skills in different grade levels in China. Overall, our findings showed that the relationship between ANS acuity and mathematics skills depends on the type of ANS aspect, the type of mathematics outcome assessed, and the grade level. Among kindergarteners, non-symbolic estimation uniquely predicted early mathematics skills, numerical operations, and mathematical problem solving. Symbolic estimation explained unique variance only in early mathematics skills. Symbolic estimation also predicted mathematical problem solving among the second- and fourth-graders, and numerical operations among the fourth-graders.

In line with our expectation, non-symbolic estimation made unique contributions to mathematics skills only in kindergarten. This replicates the findings of earlier studies, which found that non-symbolic estimation played a unique role in early mathematics skills (e.g., Clements and Sarama, 2007; Inglis et al., 2011; Desoete et al., 2012; Xenidou-Dervou et al., 2016; Starr et al., 2017). As Xenidou-Dervou et al. (2016) have noted, the start of formal mathematics education may cause symbolic estimation to become a prominent predictor of mathematics skills. It should be noted that non-symbolic estimation in kindergarten made a substantial contribution to early mathematics skills other than numerical operations and mathematical problem solving, which replicates the results of a recent meta-analysis (Schneider et al., 2017). Schneider et al. (2017) found that the correlation between non-symbolic estimation and early mathematics skills was higher than that between non-symbolic estimation and formal mathematics skills such as arithmetic. Previous studies have also shown that non-symbolic estimation correlates highly with early numerical skills such as counting and non-symbolic arithmetic (Gilmore et al., 2007; Libertus et al., 2013; van Marle et al., 2014).

Symbolic estimation made unique contributions to mathematical problem solving in Grades 2 and 4, and to numerical operations in Grade 4. The effect of number line estimation on numerical operations and mathematical problem solving is in line with the findings of previous studies (e.g., Jordan et al., 2013; Tosto et al., 2017; Zhu et al., 2017). It was surprising that symbolic estimation did not uniquely explain numerical operations in Grade 2, although it is in line with Geary (2011), who found that number line estimation in Grade 1 did not concurrently predict numerical operations. It suggests that symbolic estimation may be more important in learning more complex arithmetic such as fractions. Grade 4 students in Chinese are learning fractions (Shanghai Municipal Education Commission, 2004), and thus are handling fraction problems in the numerical operations task. Previous studies have found that number line estimation is very important in learning fraction knowledge (Jordan et al., 2013; Hansen et al., 2015), since it may provide children with an advantage in learning fraction concepts. Jordan et al. (2013) also argued that fraction knowledge may facilitate the number line estimation since children may use proportion strategies in number line task, such as mentally dividing the line into quarters to get more precise estimation (Siegler and Opfer, 2003).

In contrast to our expectation, symbolic estimation uniquely explained only TEMA-3, but not numerical operations or mathematical problem solving among kindergartners. This might be due to the fact that early mathematics tasks included items such as number comparison, and number knowledge is closely connected with the performance on number line estimation. Children in kindergarten were learning to map symbolic digits onto pre-existing non-symbolic representations (Barth et al., 2005; Mundy and Gilmore, 2009), and thus the number line estimation correlated with the early mathematics skills. Another reason may be that the early mathematics skills may promote the performance on number line tasks. Previous studies showed that young children typically use counting-based strategies when placing a number on the number line (Petitto, 1990; Schneider et al., 2008), and thus children with better counting skills may estimate more precisely on the number line task.

Symbolic estimation did not uniquely predict calculation fluency among school-age children, which was in line with the findings of previous studies (Sasanguie et al., 2013; Zhu et al., 2017). For example, Sasanguie et al. (2013) found that number line estimation among Grades 1–3 children uniquely predicted their performance on a comprehensive mathematics achievement test 1 year later, but failed to predict their performance on a timed arithmetic test. However, Zhu et al. (2017) found that number line estimation in Grade 2 and not in Grade 4 uniquely predicted concurrent calculation fluency after controlling for general cognitive abilities. A possible explanation might be that Zhu et al. (2017) did not include non-symbolic estimation in their study. An alternative explanation may be that we used the accuracy of number line estimation, while calculation fluency assessed the speed of arithmetic, which may tap on the speed of activating number representations. Holloway and Ansari (2009) found that the distance effect in a symbolic comparison task (calculated from accuracy scores of elementary children) did not correlate with calculation fluency, while that calculated from the response time scores uniquely explained calculation fluency. As Tosto et al. (2017) have argued, the limited role of symbolic estimation in calculation fluency may indicate that symbolic estimation may be less important for arithmetic once calculation reached an automatic level.

Some limitations of the present study are worth mentioning. First, the cross-sectional design of this study does not allow us to draw conclusions about the causal relationships between the two ANS aspects and mathematics skills. The direction of their relation should be examined further since recent studies also showed that mathematics skills may enhance ANS acuity (e.g., Friso-van den Bos et al., 2015). Second, we did not assess the role of home numeracy environment in our study. Previous studies have found that home numeracy environment is an important predictor of children’s mathematics achievement (e.g., Manolitsis et al., 2013; Deng et al., 2015), and the mathematics activities at home may also promote children’s non-symbolic and symbolic estimation (e.g., Mutaf-Yildiz et al., 2018). Future studies should examine the effects of home numeracy environment on ANS acuity and mathematics skills.

## Conclusion

Taken together, our results showed that the two ANS aspects have different effects on mathematics skills at different learning periods: non-symbolic estimation was uniquely related to mathematics skills in kindergarten, while symbolic estimation was uniquely related to mathematics skills in elementary school years. These results suggest that different types of ANS acuity should be used to predict mathematic skills in different learning periods and perhaps to identify children at-risk for mathematics difficulties. Moreover, interventions to promote children’s mathematics skills should target different ANS aspects for young and school-age children.

## Ethics Statement

This study was carried out in accordance with the recommendations of Ethical Guidelines for the Protection of Human Subjects of Research, Academic Ethics Committee at Shanghai Normal University. The protocol was approved by Academic Ethics Committee at Shanghai Normal University. The parents of all children gave their written consent in accordance with the Declaration of Helsinki.

## Author Contributions

DC, GG, WW, and YL designed the study. WW, DC, and LZ collected the data, prepared the data for analysis, and wrote the manuscript. GG, DC, WW, and YL revised the manuscript.

## Conflict of Interest Statement

The authors declare that the research was conducted in the absence of any commercial or financial relationships that could be construed as a potential conflict of interest.

## Footnotes

1 This is because Chinese children attend Kindergarten at the age of 3 and stay in kindergarten for 3 years before they go to Grade 1. In Kindergarten, they learn to perform simple calculations.

2 This time limit was decided based on a pilot study we conducted as well as based on the time limit used in previous studies with children of the same age as ours (e.g., Fazio et al., 2014; Libertus et al., 2016).
